# Beyond Known Barriers—Assessing Physician Perspectives and Attitudes Toward Introducing Open Health Records in Germany: Qualitative Study

**DOI:** 10.2196/19093

**Published:** 2020-11-06

**Authors:** Julia Müller, Charlotte Ullrich, Regina Poss-Doering

**Affiliations:** 1 Department of General Practice and Health Services Research University Hospital Heidelberg Heidelberg Germany

**Keywords:** eHealth, medical records, open notes, personal health records, primary health care, qualitative research

## Abstract

**Background:**

Giving patients access to their medical records (ie, open health records) can support doctor-patient communication and patient-centered care and can improve quality of care, patients’ health literacy, self-care, and treatment adherence. In Germany, patients are entitled by law to have access to their medical records. However, in practice doing so remains an exception in Germany. So far, research has been focused on organizational implementation barriers. Little is known about physicians’ attitudes and perspectives toward opening records in German primary care.

**Objective:**

This qualitative study aims to provide a better understanding of physicians’ attitudes toward opening records in primary care in Germany. To expand the knowledge base that future implementation programs could draw from, this study focuses on professional self-conception as an influencing factor regarding the approval for open health records. Perspectives of practicing primary care physicians and advanced medical students were explored.

**Methods:**

Data were collected through semistructured guide-based interviews with general practitioners (GPs) and advanced medical students. Participants were asked to share their perspectives on open health records in German general practices, as well as perceived implications, their expectations for future medical records, and the conditions for a potential implementation. Data were pseudonymized, audiotaped, and transcribed verbatim. Themes and subthemes were identified through thematic analysis.

**Results:**

Barriers and potential advantages were reported by 7 GPs and 7 medical students (N=14). The following barriers were identified: (1) data security, (2) increased workload, (3) costs, (4) the patients’ limited capabilities, and (5) the physicians’ concerns. The following advantages were reported: (1) patient education and empowerment, (2) positive impact on the practice, and (3) improved quality of care. GPs’ professional self-conception influenced their approval for open records: GPs considered their aspiration for professional autonomy and freedom from external control to be threatened and their knowledge-based support of patients to be obstructed by open records. Medical students emphasized the chance to achieve shared decision making through open records and expected the implementation to be realistic in the near future. GPs were more hesitant and voiced a strong resistance toward sharing notes on perceptions that go beyond clinical data. Reliable technical conditions, the participants’ consent, and a joint development of the implementation project to meet the GPs’ interests were requested.

**Conclusions:**

Open health record concepts can be seen as a chance to increase transparency in health care. For a potential future implementation in Germany, thorough consideration regarding the compatibility of GPs’ professional values would be warranted. However, the medical students’ positive attitude provides an optimistic perspective. Further research and a broad support from decision makers would be crucial to establish open records in Germany.

## Introduction

Giving patients access to their medical records aims at transparently informing them about their health-related data and enabling patient-centered care and shared decision making. While providing patients with access to copies of their records was already proposed in 1973 [1], the idea gained momentum since the open notes study was conducted in the United States in 2010: 105 primary care physicians encouraged more than 19,000 patients to access their medical notes [2]. According to the aim of the open notes study, open health records can be understood as a concept that includes all projects that “...provide patients with access to their medical records...” as seen on page 462 of Delbanco et al [2]. Confirmed by several studies, open access to medical records has led to (1) patients’ enhanced health literacy, (2) improved adherence to therapy, (3) increased health-related self-care, (4) improved doctor-patient communication, and (5) improved quality of care [2-9].

The German government regularly supports research projects on patient-centered care and shared decision making. However, the successful implementation of projects that aim at facilitating patient-centeredness in routine care remains an exception [10]. This also accounts for open-record concepts: in Germany, patients are entitled by law to have access to their medical records and request copies of documents that specify their medical care [11]. However, there is no comprehensive master record per patient and no structured procedure that governs how patients can access information stored in commonly used, physician-managed electronic patient files as of yet, and patients still do not automatically have access to their content. Although the first research projects on cross-sectoral personal electronic health records (PEHRs) were conducted in Germany [12-15], the patients’ unrestricted access to their records remains an exception: the patients’ right to access their records is usually solely met by printing out test results, diagnostic assessments, or related parts of their medical record upon patient request [16]. Prior research on giving patients full access to their medical records focused on implementation barriers [5,7,17-24]. Besides barriers such as data security or the patients’ potentially limited abilities to access and fully understand their records [18,20,22,24], physicians’ concerns about opening their patients’ records were recurrently described [18-21,23]. However, the reasons for physicians’ reluctance regarding such a concept remained unaddressed. Little is known, in particular, about general practitioners’ (GPs) perspectives on opening records and their perception of compatibility with the medical profession. Accomplished projects in the United States have proven that barriers can be overcome [2,7], which signals the need for understanding the perspectives of German GPs.

Opening records aims at facilitating transparency and cooperation between physicians and patients. These objectives might constitute a contrast to a GP’s professional self-conception, which is influenced by specific shared values within the medical profession. Decades ago, Eliot Freidson’s analysis of the nature of professions concluded that autonomy was the fundamental criterion that distinguished professions [25,26]. Freidson argued that professional autonomy depended on protection and tolerance for its sustainability and that the freedom from outside control was based on three claims: (1) professionals have an unusual professional skill and knowledge degree that nonprofessionals cannot evaluate, (2) professionals are responsible and may be trusted to work without supervision, and (3) the profession itself can be trusted to deal with incompetent or unethical members. His theory discussed the characterization of the medical profession by largely acknowledged autonomy and self-control, both legitimized by a knowledge monopoly accepted by society, and subsequently found broad support [27-31]. Legitimate professional autonomy provides physicians with freedom to practice their trained craft independently and to guide and instruct other health professions. This profession-defining autonomy entails professional self-control, which can be understood as freedom from external control. Both of these attributes are enabled and justified by the physicians’ unique professional knowledge that stems from their systematic, specialized medical training [25,26]. This view on the medical profession can create a strong identification with the specific values and, therefore, can influence the professional self-conception of GPs. Thus, it might affect their approval for innovating concepts like open notes as well as their perception of implications and requirements for a similar implementation in Germany.

In recent years, the change from a paternalistic to a more participatory and patient-centered relationship between physicians and patients progressed noticeably [32]. Prior research on open record concepts found that these can contribute to patient-centeredness [3,7,8]. However, these studies did not explore whether the professional self-conception of GPs in Germany is compatible with such concepts. Therefore, the aim of this study was to improve the understanding of current and future physicians’ perspectives and attitudes beyond already-known barriers. In order to identify potentially different perspectives, GPs and advanced medical students were interviewed. Based on Freidson’s theory on professional self-conception of medical doctors, perspectives of both groups were explored with a focus on the potential impact on their approval for open health records. Anticipated implications, expectations for future records, and perceived conditions for an implementation of an open record concept in German general practices were addressed.

## Methods

### Study Design

This qualitative study was conducted to explore and assess GPs’ and advanced medical students’ perspectives and attitudes toward the concept of open records and a potential implementation in Germany. Differences between the two participant groups were to be explored as well. Data were collected through semistructured guide-based interviews with GPs and advanced medical students in the Rhine-Neckar region in Baden-Wuerttemberg, Germany. The interview guide (see Multimedia Appendix 1) was discussed with a group of junior researchers (peer students of JM) in a qualitative research colloquium (led by CU and RPD) at the Department of General Practice and Health Services Research, University of Heidelberg. Adjustments were made according to recommendations. The open-ended interview questions were based on theoretical considerations and an extensive literature search. Additionally, a study-specific questionnaire was used to collect data on participant characteristics (see Multimedia Appendix 2).

For this study, ethical approval was given by the Ethics Committee of the University Hospital Heidelberg (S-529/2019). The study was reported according to the COREQ (Consolidated Criteria for Reporting Qualitative Studies) checklist for qualitative research [33].

### Participants and Recruitment

Purposive sampling was conducted without calculating a formal sample size. Structural variance was provided through diversity in age and gender. Participants were eligible for inclusion when they were (1) a resident general or internal practitioner working in a general practice or (2) an advanced medical student; further eligibility criteria were (3) a fluent command of German or English and (4) working in a location in the Rhine-Neckar region. Physicians were to be excluded when they were hospital based or specialized in another medical field. Students were to be excluded when they had not yet successfully completed their first of three medical state exams.

GP names and contact information were identified from the official medical register of the Association of Statutory Health Insurance Physicians Baden-Wuerttemberg. Medical students were identified by receiving contact information through one of their peers who acted as a gatekeeper. There were no prior relationships with any participant. Between August 21 and September 9, 2019, recruitment emails were sent to 31 GPs and 10 medical students. An information sheet with detailed background information on the aim and details of the study was attached to the email. Follow-up calls were conducted after one week. Interest was expressed by 8 GPs and 7 medical students; 1 GP withdrew interest without specification. In total, 7 GPs and 7 students participated in the study. All participants gave their written informed consent for participation and audiotaping of the interviews. The participants’ anonymity and confidentiality were ensured throughout the entire study. The participants did not receive any reimbursement for their participation in the study.

### Data Collection and Analysis

All interviews were conducted by one female author (JM) with a background in health and nursing management, health services research, and implementation science. After 12 interviews (5 GPs [42%] and 7 medical students [58%]), data saturation was reached, and data sufficiency was assessed based on deviant observations and consistency of findings. No additional themes were identified in the 2 further interviews. To accommodate participant preferences, all interviews were performed face-to-face or via telephone. Nonparticipants were not present during the interviews. No additional notes were taken during or after the interviews, and no repeat interviews were carried out.

All interviews were audiotaped, pseudonymized, and transcribed verbatim following appropriate transcription guidelines. Transcripts were not returned to participants for verification. After completion of data collection, transcripts were analyzed by the author (JM). Analysis was conducted according to thematic analysis by Braun and Clarke [34]. The identification of themes was performed deductively a priori from the interview guide (see Multimedia Appendix 1) and inductively de novo from data during the analysis. All themes were organized into main themes and subthemes. Each theme was clearly defined by a quote from the interview transcripts (see Multimedia Appendix 3). Data, derived themes and subthemes, and the analytical process were discussed regularly with supervisors (RPD and CU) and peer junior researchers during the mentioned qualitative research colloquium. The coding of transcripts was conducted in MAXQDA Standard 2018, version 18.2.0 (VERBI Software). The participant characteristics were analyzed descriptively using Microsoft Excel, version 16.28.

## Results

### Overview

All 14 interviews were conducted between August 28 and September 25, 2019. GP interview durations ranged from 14 to 31 minutes (mean 20, SD 6), and student interview durations ranged from 14 to 42 minutes (mean 28, SD 11). To accommodate a GP request, 1 interview was performed face-to-face in a public café, while all other interviews were conducted via telephone. All student interviews were held face-to-face. Out of the 7 students, 4 were interviewed in a seminar room at the University of Heidelberg and the remaining 3 at JM’s private domicile to accommodate participant preferences. Participant characteristics were collected from all interviewees. The age of GPs ranged from 40 to 60 years (mean 50, SD 8) and the age of students ranged from 22 to 26 years (mean 24, SD 2). A total of 4 GPs out of 7 (57%) and 3 out of 7 participating students (43%) were female. Table 1 provides further information on the participant characteristics.

**Figure 1:**
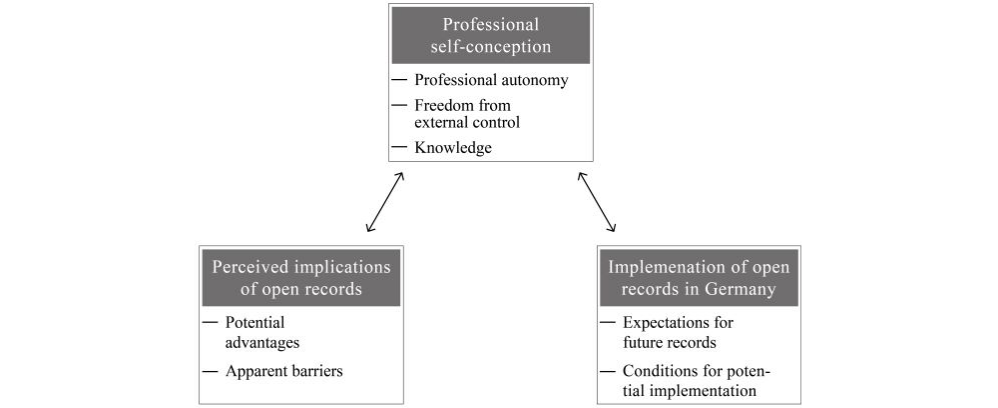
Overview of identified themes on participant perspectives and attitudes regarding open health records in this study.

The key findings of this qualitative study reflect the participating GPs’ and advanced medical students’ perspectives and attitudes and are presented with a focus on three main themes and associated subthemes identified from the data (see Figure 1). When applicable, themes were differentiated by participant groups. All provided interview quotes indicate the respective participant group and transcript position (TP) and were translated from German into English with due diligence. To transparently indicate the distribution of themes across interviews, their participant designation is also provided.

**Table 1:** Participant characteristics (N=14).

Characteristic			Value, n (%) or mean (SD), range
**General practitioners (n=7)**			
	Professional specialization: general practice, n (%)		7 (100)
	Years of practice, mean (SD), range		23 (8), 11-34
	Age in years, mean (SD), range		50 (8), 40-60
	**Gender, n (%)**		
		Female	4 (57)
		Male	3 (43)
**Students (n=7)**			
	Practical experience: medical traineeship in general practice, n (%)		7 (100)
	Years of study, mean (SD), range		5 (1), 4-6
	Age in years, mean (SD), range		24 (2), 22-26
	**Gender, n (%)**		
		Female	3 (43)
		Male	4 (57)

### Professional Self-Conception and its Effect on Attitudes Toward Open Records

Focusing on the GPs’ reluctance, the concept of giving patients full access to medical records emerged to be incompatible with the GPs’ professional values. As a first aspect of their professional self-conception, GPs considered their *professional* *autonomy* an essential professional value. They advocated for maintaining their professional independency (GPs 2, 8, and 11-13) and voiced that opening records for patients would make them feel restricted in their profession (GPs 2 and 11-13).

Because we need a certain...freedom to do our job, that nobody has access to our own things. ...That would damage too much of our medical profession as we see it.

GP 12: TP 249-253

I write quite delicate things in my records which, as I said, should not be read by people.

GP 2: TP 261-262

It was important to the GPs that certain parts of the patient record, especially their additional personal notes of perceptions that go beyond clinical data, were to be kept confidential (GPs 2, 3, 8, and 11-14). They emphasized their autonomy by explaining that records were not meant for patients but rather for themselves. Even though GPs stated that they had already given patients access to their medical records by printing parts of them out on request (GPs 2, 3, 8, 11, 12, and 14), they repeatedly labelled the medical record as their personal property. This position was expressed in a rather possessive language, whereby GPs characterized the medical records as *my personal records* (GP 11: TP 39), *a DIARY for me* (GP 13: TP 126), or *MY fundamental right* (GP 12: TP 266).

I want MY notes and MY things for myself.

GP 11: TP 87

The GPs’ aspiration for autonomy was also assessed by the medical students. Based on their experience from internships, they emphasized that GPs, especially, were used to working very self-sufficiently and that they insisted on the independency of the medical documentation (Students 1, 6, and 10). Moreover, it was mentioned that GPs would not want to share the entire patient record unconditionally (Student 6). Referring to their practical experiences, the students confirmed that GPs provided parts of the record on request. However, they emphasized that asking for access can be a barrier for patients and that, as a result, those requests rarely occurred (Students 1 and 10).

But there is a therapeutic privilege...that we don’t always say everything, and I think many GPs want to keep that...

Student 6: TP 298-300

In contrast to the GPs’ demand for autonomy, the students voiced a rather cooperative attitude when addressing their own professional behavior: they emphasized that not being the sole owner of the patients’ data might facilitate shared decision making with patients (Students 1, 4, 7, and 10), support interprofessional exchange (Students 1, 6, 7, 9, and 10), and, therefore, improve quality of care (Students 1, 6, 7, 9, and 10).

Besides professional autonomy, the participating GPs insisted on their right for self-control, which was referred to as *freedom from external control*. By opening records, they anticipated being controlled by patients or third parties (GPs 2, 8, and 11-13). They highlighted that they would not want to be confronted with potential mistakes or divergent opinions, and they would, furthermore, not want to be forced to discuss and defend their decisions (GPs 2, 3, 8, and 11-13).

It is not acceptable that patients have insight and...confront me with things they have read in the record.

GP 8: TP 56-58

Confirming this, the students also reported self-control as a significant factor for GPs. Referring to their experiences from traineeships, they indicated the undesirability of external control for GPs (Students 1, 6, 7, and 9). They voiced that GPs did not want to explain their professional behavior and that giving patients access to records would make GPs feel pressured to justify themselves and their actions (Students 6 and 7). Contrary to their perception of the GPs’ attitudes, the students valued the patients’ engagement and viewed the potential control as positive for their own profession: by encouraging patients to access their medical records, they expected themselves to reflect more on their medical actions (Students 1 and 7) and to be able to work more closely together with patients (Students 1, 4-6, and 10). Again, they assumed an increase in quality of care as a potential result (Students 1, 6, 7, 9, and 10).

I think the biggest chance is that you have to reflect on yourself and that you are able to do that. What you might not do if you just type it in.

Student 1: TP 120-122

Besides professional autonomy and freedom from external control, the GPs’ *knowledge* was voiced as an aspect of their professional self-conception. The GPs emphasized that patients needed to trust the GPs’ assessment due to their medical expertise and practical experience (GPs 2, 8, 12, and 13).

...the patient has to accept that the doctor knows the field of expertise better.

GP 12: TP 166-167

Doctors considered themselves to function as a professional filter that, based on their medical knowledge, needed to screen all information for patients (GPs 2, 12, and 13). Both the superiority of knowledge and the GPs’ filter role were also indicated by the students who underlined the GPs’ expertise with the length of their medical studies (Students 1, 4, 5, and 9). Another aspect was shared when the perceived power of knowledge was voiced: few students hypothesized that by giving patients access to their records and encouraging them to inform themselves, GPs might feel like they would be losing intellectual advantage and power (Students 1 and 6).

And I think it deprives the doctor of some of the power asymmetry he has due to his supposed knowledge.

Student 6: TP 179-181

However, for their own profession, the students anticipated the knowledge asymmetry between them and patients to decrease by opening records. Moreover, they demanded this in order to encourage the patients’ acquisition of medical knowledge (Students 1, 6, and 9).

GPs assumed that the students’ acceptance of open records would be higher than their own and that this potential openness might stem from their lack of practical experience. It was also assumed that as students become increasingly enculturated through practice, they would be more reluctant as well (GPs 2, 3, 8, and 11-13). The students saw the GPs’ reluctance to change as a potentially obstructive factor to their approval for open records (Students 1, 4, 7, and 9). They anticipated the GPs’ concern of losing their professional autonomy and the freedom from external control as potential reasons for a lower acceptance (Students 1 and 6).

### Perceived Implications of Open Records

When contemplating open health records, potential advantages and apparent barriers were commonly mentioned. Among the potential advantages, *patient education and empowerment* were anticipated by both participant groups: both students and GPs expected that patients would be better informed about their health. They mentioned that reading their medical records might encourage patients to increase their health literacy (GPs 2, 3, and 12-14; Students 1, 6, 7, 9, and 10). Furthermore, the patients’ adherence to treatments (GPs 2, 12, and 14; Students 1, 4-7, and 10) and their health-related self-responsibility (GPs 2, 3, and 14; Students 1, 4-7, 9, and 10) were assumed to increase. Moreover, besides these patient-related aspects, GPs and students anticipated a *positive impact on the practice*: they emphasized the advantage of sharing results between disciplines if patients could authorize other specialists to access their records (GPs 2 and 8; Students 1, 6, 7, 9, and 10). By providing patients with the innovative functions of open records, few participants envisioned a market advantage (GP 8; Students 6 and 7). A higher *quality of care* was expected by students only. They assumed the quality of care would increase when detecting errors and improving diagnoses through the cooperation with informed patients (Students 7, 9, and 10).

Besides the potential advantages, the GPs and medical students assessed barriers. Among these, *data security* appeared to be an essential problem, voiced by all participants but one (GPs 3, 8, and 11-14; Students 1, 4-7, 9, and 10). When voicing this barrier, a contrast occurred between GPs and students: while GPs highlighted the demand for data protection, students rather assessed the fear of data abuse through hacking as a barrier, which they characterized as a particular aspect of the “German mentality” on data security. Besides data security, participants anticipated an *increased* *workload* when opening records for patients: although few participants were convinced that the workload would, if at all, only change at the start of an implementation phase (GP 14; Student 9), others expected patient requests to rise and the duration and number of patient contacts would increase permanently (GPs 2, 3, 8, and 11-13; Students 1, 4, 7, and 10). A higher workload was, furthermore, anticipated due to an intensified documentation effort in order to avoid misunderstandings (GPs 8, 12, and 14; Students 1, 6, 7, 9, and 10).

...patients think they understand it, but then a small note in the record would cause a misunderstanding. That would be disastrous. It would cost us an incredible amount of time and effort to resolve it.

GP 3: TP 111-114

As a consequence of the increased workload, few participants expected the practice’s *costs* to increase. Furthermore, they assumed that these costs would not be reimbursed (GP 2; Students 6 and 7). Referring to the *patients’ limited abilities*, GPs emphasized the patients’ insufficient medical understanding (GPs 2, 3, 8, and 12) and the risk of provoking anxiety (GPs 2, 3, 12, and 13). A more optimistic view was reported by students: although they saw the risk of provoking anxiety as well (Students 1, 4, 7, and 10), they considered the patients’ abilities sufficient for understanding their records. However, older age and the burden of disease were identified as limiting factors for the patients’ understanding among students and GPs alike (GPs 2, 8, 11, 13, and 14; Students 4, 7, and 9). As another barrier, the *physicians’ concerns* were reported by GPs and students. While GPs justified their hesitance with the mentioned barriers (GPs 2 and 14), the students assessed the GPs’ reluctance to change and their professional self-conception as obstructing factors (Students 1, 6, 7, and 10).

### Implementation of Open Records in Germany

Most students considered the implementation of open health records in German general practices to be essential and realistic within 10 years (Students 1, 5-7, 9, and 10). GPs could only imagine the implementation of *partly accessible records* in which GPs’ additional personal notes, which go beyond clinical data, are kept nonaccessible to patients (GPs 2, 8, and 12-14). Besides the idea of open notes, GPs and students both anticipated future records in general practices to turn entirely *electronic* (GPs 2, 3, and 11-14; Students 1, 4-7, 9, and 10). Furthermore, they hoped for a *cross-sectoral compatibility* to facilitate the requests of findings between medical specialists from different sectors who are involved in a patient’s care (GPs 3, 8, 12, and 13; Students 1, 6, and 7).

For implementation in Germany, *reliable* *technical conditions* were requested: the participants insisted on the security (GPs 3, 8, and 12-14; Students 1, 4-7, 8, and 10) and reliability (GPs 2, 8, and 14; Students 1, 4, 6, 7, 9, and 10) of a future system. Furthermore, participants suggested a translation program for medical terms to provide patients with easily comprehensible translations of medical jargon (GPs 3 and 8; Students 5 and 6). A strong emphasis, voiced by the GPs, was put on the availability of a “black box” within the system that enables them to keep additional personal notes, that go beyond clinical data, private (GPs 2, 8, and 12-14). Referring to the *participants’ consent*, the students advocated for the patients’ informed consent (Students 1, 5-7, 9, and 10), while the GPs indicated their own approval as essential (GPs 2, 11, and 12). Advocating for a comprehensive change of systems, few students proposed an obligatory implementation (Students 7 and 10), while GPs emphasized that this would lead to strong resistance (GPs 2 and 11). Therefore, the participants demanded the *GPs’* *involvement* in the development of the implementation project; for instance, providing reimbursement for participation (GP 2; Students 6, 7, and 10) and incorporating the GPs’ demands (GP 2; Student 1) were considered mandatory.

## Discussion

### Principal Findings

The aim of this study was to explore GPs’ and advanced medical students’ perspectives and attitudes toward open health records. All barriers found in this study were reported by prior studies as well when addressing the general introduction of electronic health records [35,36] and when investigating physicians’ perceptions on open notes implementations [17-20,23]. Although the reluctance of physicians is a frequently reported barrier, evaluation studies of former implementation projects showed that the physicians’ initial concerns diminished after participating in pilot projects [2,19-21,37]. Also, the GPs’ concern of an increased time effort has been disproven in the past [4]. The potential advantages discovered in this study were correspondingly reported when exploring physicians’ expectations before participation [17-20,23] and when investigating effects of giving patients access to records [2,4]. Although advantages were confirmed and barriers were overcome in former studies with PEHRs in Germany [12-15], this study found that the GPs’ perspectives on open records were characterized by hesitation rather than by optimism.

Findings of this study indicate that the GPs’ professional self-conception as an underlying attitude can obstruct their approval for open records. GPs repeatedly emphasized their perception of professional values being threatened by opening records. Furthermore, the medical record was repeatedly referred to as belonging to the GPs rather than as the patients’ property. Affirming previous findings [23], physicians strongly advocated for keeping the patient record within the professional group of medical doctors. When GPs were open toward sharing parts of the record, they primarily referred to the patients’ option of requesting printouts instead of accessing them directly. In contrast to this, patients in Germany are entitled by law to get access to their medical records [11], and former research such as the open notes studies [3] or research on PEHRs in Germany [20] has shown that patients indeed want to access their records. According to the student participants in this study, having to ask for printouts is, however, perceived as a barrier for patients, which was also found in previous research [16,19].

Although some GPs considered diagnoses or test results to be objective and would, therefore, give patients access to these, all of them strongly refused to disclose additional notes they take for their eyes only. This corresponds to prior research [2,21], which found that physicians were reluctant when they were asked to share their notes with patients. When GPs referred to the records as their property, a paternalistic attitude became apparent, which might be influenced by the German health care environment in which patient-centered concepts still remain an exception in routine care, even though they are considered beneficial [10].

The medical students’ experiences with GPs confirmed the professional self-conception as a determining factor: they highlighted that the work of GPs was especially characterized by autonomy and freedom from external control, and they empathized with the GPs about their perceived loss of these values. However, the students’ aspiration for enabling shared decision making through open notes became apparent: they saw the concept as a way to improve their own professional behavior and achieve optimized care for patients, and they emphasized that the advantages would outweigh the effort of overcoming barriers. Although they agreed that GPs have advanced knowledge, they advocated for reducing the asymmetry of knowledge and information between GPs and patients. While there is a shortage of research on medical students’ perspectives on open notes, the positive attitude toward patient empowerment has been reported before [38].

The GPs’ demanding attitude of acting as autonomous, self-controlled, and knowledgeable professionals and the medical students’ striving for engaging patients through open records show a strong contrast. According to the GPs, this divergence might stem from the students’ lack of practical experiences or even generational differences. It was reported before that professional values are developed and shaped by the social setting in which first practical experiences are gained during and after medical training [25,26,39]. Furthermore, research indicates that students of the current generation are generally open to using digital technology for their own health-related matters [40]. However, whether these are the main reasons for the students’ evident optimism on open records remained unclear. The students indicated that the GPs’ hesitance was caused by their desire for professional autonomy and their reluctance to change, which appeared to originate in the aspiration for maintaining their professional values. Some of the GPs’ rather paternalistic perspectives correspond to the “doctor knows best” literature: prior research found that doctors, as well as patients, had difficulties in putting a less paternalistic way of communicating into practice and move toward shared decision making in routine care [41]. A systematic review on health care providers’ perspectives on shared decision making showed that a lack of agreement with the concept was one of the main barriers for its implementation [42]. Even though the shift from paternalistic to participatory medicine gained momentum in the past decades [32], the physicians’ professional self-conception still seemed to correspond to the traits discussed by Freidson [25,26]. While these studies support some of the current findings, there is still a lack of studies on physicians’ professional self-conception. Besides the fundamental research on medical professions [25,26,31], no recent studies were identifiable. Although few studies found that physicians perceived a loss of autonomy when patients became more knowledgeable [43] or addressed the issue of merging the physicians’ aspiration for autonomy with the concept of informed decision making [44], research on GPs’ professional values and their compatibility with open health records is still pending. Even though identified conditions for an implementation of open records were reported previously [45-47], considering the physicians’ attitudes based on their professional self-conception has not yet been researched.

### Strengths and Limitations

This study focused on the underlying attitude of GPs and medical students toward the concept of sharing medical records with patients in German general practices. In contrast to previous studies, which mostly consisted of describing implementation barriers and enablers, this study provided a perspective beyond these already-known aspects as the first of its kind. The qualitative design allowed an in-depth view on the GPs’ professional self-conception, which influenced their approval for open records. This focus highlighted a previously unaccounted fundamental barrier for the introduction of open records in the German context. Furthermore, this study incorporated a balanced sample of both GPs and advanced medical students. By providing an internal and external perspective by GPs and medical students, the findings were enriched and strengthened. Moreover, structural variance was accomplished through a balanced distribution in age and gender.

Some limitations must be acknowledged. In this study, only the Rhine-Neckar region in Germany as one geographic area was focused on. Specific national and regional factors might have influenced the results. By focusing on general practices, only one medical specialty was addressed. Both considerations might limit a general transfer of findings. The recruitment of medical students was facilitated through the use of a gatekeeper. Therefore, the sample might represent a positive selection of medical students as well as of GPs who might have participated due to their general interest in open records. Therefore, results must be interpreted with caution. Even though data saturation was reached, a higher number of participants could have led to more diverse results. All quotes were translated from German into English with due diligence. However, it is possible that fine linguistic characteristics in the translated quotes differ from the original German quotes. Furthermore, although different perspectives were ensured by discussing the study in a qualitative research colloquium, the interviews and analyses were only carried out by one researcher.

### Conclusions

Giving patients access to their medical records can increase transparency in health care. Compatibility with physicians’ professional values and their acceptance is crucial for a successful potential implementation of open health record concepts in Germany. The medical students’ commitment to engaging patients and accomplishing shared decision making provides an optimistic view. However, further research and broad support from decision makers is necessary.

## Appendix

Multimedia Appendix 1 Translated interview guide.

Multimedia Appendix 2 Translated participant questionnaires.

Multimedia Appendix 3 Definition of themes.

## Abbreviations

COREQ: Consolidated Criteria for Reporting Qualitative Studies
GP: general practitioner
PEHR: personal electronic health record
TP: transcript position
