# Supplementary material for: Beyond Known Barriers—Assessing Physician Perspectives and Attitudes Toward Introducing Open Health Records in Germany: Qualitative Study
Source: J Particip Med. 2020 Nov 6;12(4):e19093. doi: 10.2196/19093 (PMC7679209; doi:10.2196/19093)
Supplement: Multimedia Appendix 1 [file jopm_v12i4e19093_app1.pdf]

Interview guidelines

Interview Guideline for General Practitioners

Attitude towards Open Records

1. *In Germany, first research projects on transparent patient files are currently taking place. The origin of these concepts lies in the USA: In 2010, a project was realized in which more than 100 physicians gave 19,000 patients an insight into their medical records. It aimed at facilitating open communication with patients about all diagnoses, prescriptions, or the physicians' notes. The concept improved the patients' understanding and treatment adherence and patients were better prepared for visits. Furthermore, there were fewer errors in the records and the interdisciplinary cooperation improved. 99% of all patients said they wanted to continue accessing their medical records.*
   a. What is your first impression of this concept?
   b. Compared to your own documentation manner: What do you notice about this concept?
   c. What chances do you see for GPs?

2. What conditions must be fulfilled for this concept to function in Germany?

3. Let's assume that in Germany this concept existed as a pilot project:
   a. What would be the effects on communication with patients?
   b. What (further) advantages and disadvantages do you see?
   c. What would be the criteria for you to participate in the pilot project?

4. What do you think...
   a. ...how your medical colleagues assess the concept?
   b. ...how current medical students assess the concept?

5. What will the medical record look like in 10 years?

Conclusion

- What other thoughts would you like to share with me?
- Which questions do you still have?

Thank

Thank you very much for your interesting insights and your willingness to participate in the interview.

Attitude towards Open Records

1. *In Germany, first research projects on transparent patient files are currently taking place. The origin of these concepts lies in the USA: In 2010, a project was realized in which more than 100 physicians gave 19,000 patients an insight into their medical records. It aimed at facilitating open communication with patients about all diagnoses, prescriptions, or the physicians' notes. The concept improved the patients' understanding and treatment adherence and patients were better prepared for visits. Furthermore, there were fewer errors in the records and the interdisciplinary cooperation improved. 99% of all patients said they wanted to continue accessing their medical records.*
   a. What is your first impression of this concept?
   b. Compared to the forms of medical documentation you have come across: What do you notice about this concept?
   c. What chances do you see for GPs?

2. What conditions must be fulfilled for this concept to function in Germany?

3. Let's assume that in Germany this concept existed as a pilot project:
   a. What would be the effects on communication with patients?
   b. What (further) advantages and disadvantages do you see?
   c. What would be the criteria for you to participate in the pilot project?

4. What do you think…
   a. … how other medical students assess the concept?
   b. … how general practitioners assess the concept?

5. What will the medical record look like in 10 years?

Conclusion

- What other thoughts would you like to share with me?
- Which questions do you still have?

Thank

Thank you very much for your interesting insights and your willingness to participate in the interview.
