# Supplementary material for: Beyond Known Barriers—Assessing Physician Perspectives and Attitudes Toward Introducing Open Health Records in Germany: Qualitative Study
Source: J Particip Med. 2020 Nov 6;12(4):e19093. doi: 10.2196/19093 (PMC7679209; doi:10.2196/19093)
Supplement: Multimedia Appendix 2 [file jopm_v12i4e19093_app2.pdf]

Questionnaires for participant characteristics

Questionnaire for General Practitioners

Interview No.: __________

Personal Details:

Gender:

- ☐ female
- ☐ male
- ☐ diverse

Age: __________

Occupational Details:

Professional specialization: ______________________________

Type of employment:

- ☐ in own general practice
- ☐ employed in general practice

Years of practice: __________

Questionnaire for Medical Students

Interview No.: __________

Personal Details:

Gender:

- ☐ female
- ☐ male
- ☐ diverse

Age: __________

Occupational Details:

Study semester: __________

Medical traineeship and/or internship in general practice completed:

- ☐ yes
- ☐ no

Planned establishment as general practitioner:

- ☐ yes
- ☐ no
- ☐ I do not know it yet
