# Supplementary material for: Beyond Known Barriers—Assessing Physician Perspectives and Attitudes Toward Introducing Open Health Records in Germany: Qualitative Study
Source: J Particip Med. 2020 Nov 6;12(4):e19093. doi: 10.2196/19093 (PMC7679209; doi:10.2196/19093)
Supplement: Multimedia Appendix 3 [file jopm_v12i4e19093_app3.pdf]

Definition of themes

Theme: Perceived Implications

Subthemes	*Definition*	*Text samples from transcripts*
Potential advantages	Participants report anticipated chances of giving patients access to records	*Hm, chances are that they [patients] will take better care of themselves.* [GP 14: 64]
		I can imagine that maybe some conversations with sophisticated people would be more on an equal level. [GP 3: 99-100]
		I think you have the chance to have a better relationship with the patients and also to get a better connection to the patients […]. [Stud 1: 114-116]
Apparent barriers	Participants report anticipated barriers that can occur when giving patients access to records	*If someone can look into the data, then anyone else can do that too. I think this is a huge problem.* [GP 3: 68-70]
		And for someone who doesn't understand so much in this [medical] area, that maybe […] things will be misunderstood, and the patient then has to deal with it, or things he doesn't understand and then maybe becomes anxious. [GP 12: 117-120]
		[...] you also need a lot of time, especially the first time, to explain what everything means. [Stud 10: 148-150]

Theme: Professional self-conception

Subthemes	*Definition*	*Text samples from transcripts*
Autonomy	Participants report their aspiration for freedom to practice the trained craft independently	*Because we need a certain […] freedom to do our job, that nobody has access to our own things. And not only from us, but also from our patients. That would damage too much of our medical profession as we see it. And of our doctor-patient relationship in the end.* [GP 12: 249-253]
		I don't want to be in this anymore if everything is transparent and I have to disclose everything, […]. I want my profession to be a little individual. [GP 11: 200-202]
		But there is a therapeutic privilege [...] that we don't always say everything, and I think many GPs want to keep that, that they have a backstage. [Stud 6: 298-300]

Freedom from external control	Participants report their aspiration for freedom from external control	*It is not acceptable that patients have insight and [...] confront me with things they have read in the record.* [GP 8: 56-58]<br>*Why are so many colleagues against it? You would reveal everything, also the nonsense that you produced.* [GP 2: 47-48]<br>*The GP does not want to justify why he did that, in the documents. He just wants to say 'I ordered this' [...].* [Stud 6: 86-88]
Knowledge	Participants report the importance of their medical knowledge	*[...] the patient has to accept that the doctor knows the field of expertise better.* [GP 12: 166-167]<br>*It's a little exaggerated, but this is unfiltered knowledge. Even if it was completely correct, if I was completely correct. I cannot just transport that in everyday life. [...] And I think it cannot be that everything, all knowledge that I have about the patient, also from his history, which I – as an expert – write down relatively unfiltered and briefly, [can be accessed by the patient].* [GP 2: 71-78]<br>*So that the patient only informs himself on the basis of the file [...]. And then I would lose this filter, which I – as a doctor – have.* [Stud 1: 226-227]

Theme: Implementation in Germany

Subthemes	*Definition*	*Text samples from transcripts*
Expectations for future records	Participants report assumed attributes of medical records in ten years	*I do believe that it is going in this direction, that, in future, we will just use this electronic patient card, patient record.* [GP 13: 204-206]<br>*So, I think it will definitely be [...] digitized.* [Stud 4: 173]
Conditions for an implementation	Participants report conditions for Open Records in Germany	*From a data protection perspective, it should be safe in any case, so that not everyone suddenly has access to it.* [GP 14: 87-88]<br>*Well, the patients would have to agree, I guess.* [Stud 5: 154]

Theme: Miscellanea

Subthemes	*Definition*	*Text samples from transcripts*
Record provision on request	Participants refer to providing patients with records on request	*[...] so if a patient says he wants to see his file, we print it out. From this point of view, we are actually relatively open.* [GP 3: 32-34]
		I mean of course one can gain insight [on request], but this hurdle is very high for the typical patient. [Stud 1: 44-45]
Perception of others' acceptance	Participants assess the opinion of the other participant group	*[The students see it] rather positive, because nowadays everything is done with openness and discussion.* [GP 12: 214-215]<br>*I'm sure they [students] like it (laughs). Because they still see it more from the patient's point of view and they cannot at all assess the consequences in the medical everyday life yet, because they do not have the practical experiences yet. I could imagine that they are more open towards it, but because of a lack of experience.* [GP 3: 147-150]<br>*They [the GPs] see it generally certainly more critical. Because the average GP is [...] probably not so keen on changing things.* [Stud 7: 284]
